# The Bionics Bus for Neurology and Neuropsychiatry: Concept Development and Validation

**DOI:** 10.1049/htl2.70008

**Published:** 2025-03-20

**Authors:** Christopher James, Sarang Shankar, Samuel J. Tromans, Richard Laugharne, Paraskevi Triantafyllopoulou, Maria Richards, Rohit Shankar

**Affiliations:** ^1^ Biomedical Engineering Institute University of Warwick Warwick UK; ^2^ Truro School Truro UK; ^3^ SAPPHIRE Group Department of Population Health Sciences College of Life Sciences University of Leicester Leicester UK; ^4^ Adult Learning Disability Service Leicestershire Partnership NHS Trust Leicester UK; ^5^ CIDER University of Plymouth Peninsula School of Medicine Truro Truro UK; ^6^ Department of Intellectual Disability Neuropsychiatry Research Team Cornwall Partnership NHS Foundation Trust Truro UK; ^7^ Tizard Centre University of Kent Canterbury UK

**Keywords:** biomedical communication, biomedical education, health care, patient care, patient diagnosis, patient monitoring, patient rehabilitation

## Abstract

Healthcare delivery in the United Kingdom is increasingly becoming a challenging issue where demand is regularly outstripping availability. This is particularly a challenge in neurology and neuropsychiatry, where delays in diagnosis and treatment are leading to worse health and social outcomes. The Darzi report, which focused on three key tenants, has been hailed as the future blueprint for National Health Service (NHS) sustainability and high‐quality care delivery. These three tenants are moving from analogue to digital approaches, focusing on prevention and wellbeing, and supporting diagnosis and treatment in communities instead of hospitals. Technological interventions are relevant at all stages of these care pathways. There is an opportunity to identify an easy to use community‐based mobile resource to help screen, triage and refer suspect neurology and neuropsychiatric presentations to the right support. The potential benefits to patients, clinicians, organisations and communities could be significant. To enable this vision, the concept of Bionic Bus (https://bionicsbus.org/) was developed. This study looked to understand the acceptability, utility and scope of the Bionics Bus concept among the public using mixed‐methods research techniques. Results suggest high acceptability, utility and wide scope. This study gives a template for similar evidence‐based innovation to be applied for other health conditions.

## Graphical abstract

Healthcare delivery in the UK is increasingly becoming a challenging issue where demand is regularly outstripping availability. The Darzi report focused on three key tenants for the NHH i.e., moving from analogue to digital approaches, focusing on prevention and wellbeing, and supporting diagnosis and treatment in communities instead of hospitals. To enable this vision, the concept of Bionic Bus (https://bionicsbus.org/) was developed and tested among the public using mixed‐methods research techniques.

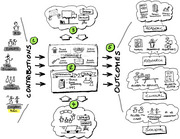

## 1 Introduction

Neurological and neuropsychiatric disease is the leading cause of disability in 2021, affecting 43.1% (95% confidence interval 40.5‐45.9) of the global population [1]. One in six people across the United Kingdom are affected by a neurological condition [1]. Their care uses some 14% (£29 billion in 2020/21) of the social care budget and 3.5% (£6.3 billion in 2020/2021) of the National Health Service (NHS) budget. Around £750 million is spent on urgent and emergency care of neurological conditions, with a 3.6% growth of this figure year on year [2]. These figures are only set to increase with an ageing population, as the prevalence of disabling neurological disorders increases steeply with age [1].

The United Kingdom has 1.1 consultant neurologists per 100,000 people, while the average in Western Europe is 8.3 [3]. Similarly, there are 17 psychiatrists per 1,000,000 in the European Union compared to 8 in the United Kingdom [4]. This is further exacerbated by regional inequalities in health care [5]. Interrogation of English GP registry data showed that epilepsy, dementia, mental health problems and stroke were all drastically over‐represented in coastal regions [6]. Another report highlighted the higher incidence of neurological disease in rural areas, with isolated geographical location, healthcare workforce recruitment challenges, social detachment and lack of transport as driving factors of poor health [7]. Deficits in neuropsychiatric health care are driven and exacerbated by regional inequalities in health care, particularly as a lack of expertise at smaller district general hospitals and community services is acknowledged to lead to inequities in the distribution of high‐quality care [8]. Yet the distribution of UK neurological and psychiatric specialists is concentrated in urban centres [3]. As a result, it is estimated that 39% of deaths of those with neurological diseases are premature [9].

Targeted rehabilitation can positively impact people's function and quality of life; however, widespread limitations in staff and resources are major barriers [10]. This presents an impending time bomb, with health and social care costs set to rise exponentially [11]. Delays in care and lack of timely interventions can have a significant impact on individuals’ mental well‐being and quality of life. It can lead to issues of unemployment and increase the risk of becoming chronically disabled. Targeted screening to direct to suitable sub‐specialties can positively impact on people's function and quality of lives, however widespread limitations in staff and resources are major barriers. Innovative, cost‐effective approaches, such as the use of technology, are needed to deal with this crisis, and alongside this, a research agenda shaped to best inform practice [12]. Technological innovation can increase productivity and replace processes that at present are reliant on clinical observations of trained healthcare professionals.

### 1.1 Patients are not Sufficiently Involved or in Control of Their own Care

Another key factor impacting neurological disease care is a lack of patient input into their own care. This is essential since people with long‐term conditions are likely to spend less than 1% of their time in contact with health professionals [13]. Patient‐led care has been shown to deliver improvements in patient satisfaction, mental health outcomes and cost [14]. Keeping this in mind the latest reports on the health of the NHS, such as the Darzi report highlight the pressing and urgent need to (a) Move care provision closer to people's homes in the community more than the hospitals, (b) enable prevention models to assist rapid screening, diagnosis and recovery, and (c) use digital and technological advancements, particularly develop assistive technology to help deliver 1 and 2 [15]. Creating care pathways that are dynamic and responsive to patient needsand empowering patients to take control of their own care has therefore become a key priority for the NHS [15].

### 1.2 The Bionics Bus Project

A description of the Bionics Bus is provided in (Appendix 1). Technological interventions are relevant at all stages of these care pathways. Given the significant challenges neurological and neuropsychiatric conditions pose, there is an opportunity to identify an easy‐to‐use, community‐based mobile resource to help screen, triage and refer suspect presentations to the right support. The potential benefits of such a resource to patients, clinicians, organisations and communities are summarised in Table 1.

**TABLE 1:** Potential care pathway benefits of the Bionics Bus.

Patient and family/carer	Clinician	Organisation	Community/State
Early screening and placement on the correct pathway.	Higher likelihood of getting correct referrals.	Improved waiting times.	Reduced risk of losing job, going off long term sick, becoming dependent on state befits, loss of skills.
Reduced stress and better psychological wellbeing.	Improved targeted testing.	Reduced costs especially expensive ones like MRI scans etc.	Improved social and health outcomes.
Sooner access to the correct management and treatments.	Improved and earlier diagnosis.	Reduced misdiagnosis and harm.	Cost reduction to state, improved productivity and wealth generation.

**FIGURE 1:**
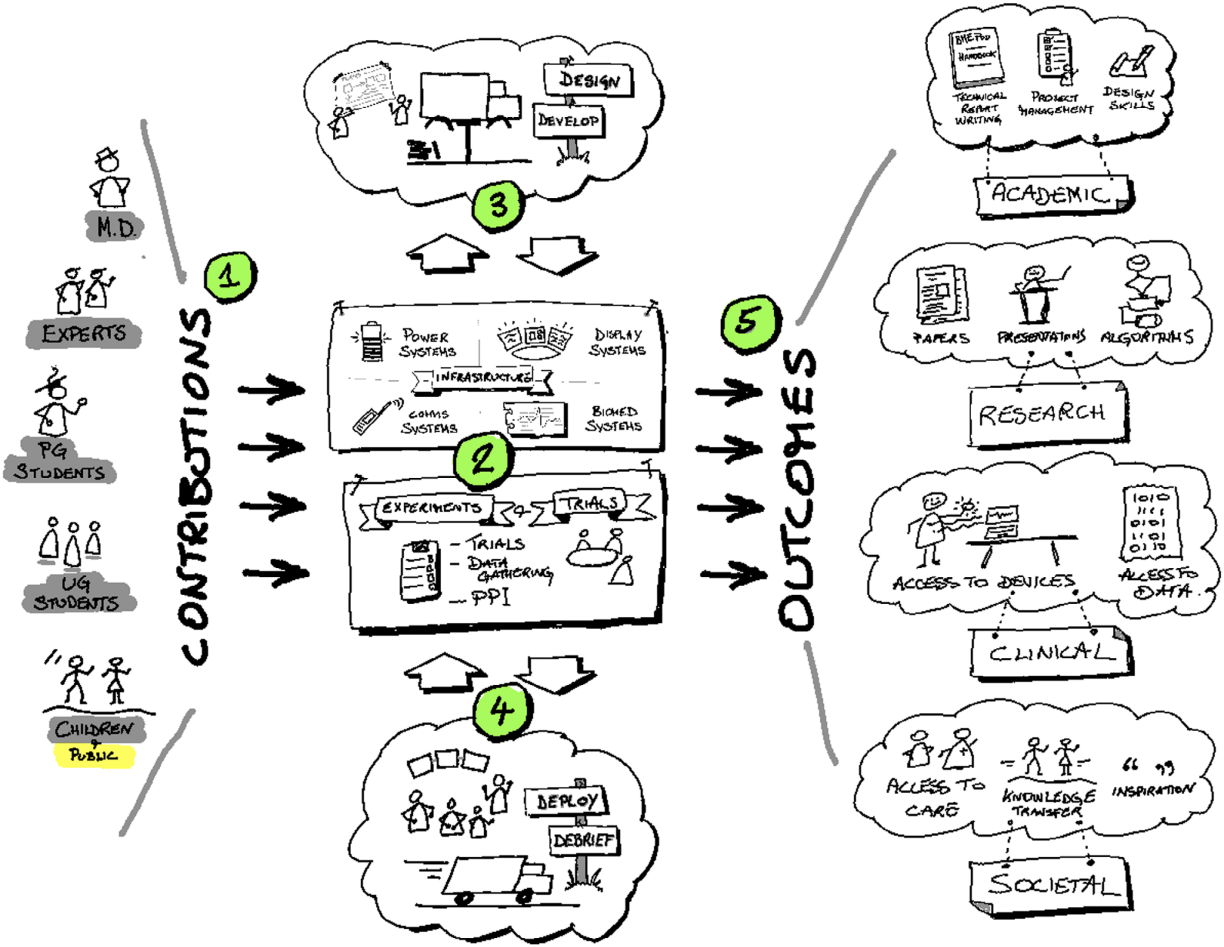
The Bionics Bus project concept/design. *This figure shows (1) the various people contributing to and benefitting from the Bionics Bus. The major activities involved (2) creation of the Bus infrastructure as well as then the creation of experiments and trials to run in the Bus. One of the major benefits of the Bus is (3) the Design and Development aspect of the work, this is then followed up (4) by the Deployment of the experiments/ trials as well as the extraction of information/ reporting from the data gained. The overall outcomes (5) are split shared between academic, research, clinical and societal outcomes*.

The Bionics Bus project is about creating a mobile platform to deliver care awareness and care to all of the stakeholders highlighted in Table 1. The platform is such as to benefit (a) those designing and performing the outreach, as well as those on the receiving end of the outreach activities; and (b) people who may benefit from technological innovations in their treatments and care who are marginalised mainly due to geography [16]. Figure 1 highlights the bus's proposed concept, design and key lines of delivery and engagement.

**FIGURE 2:**
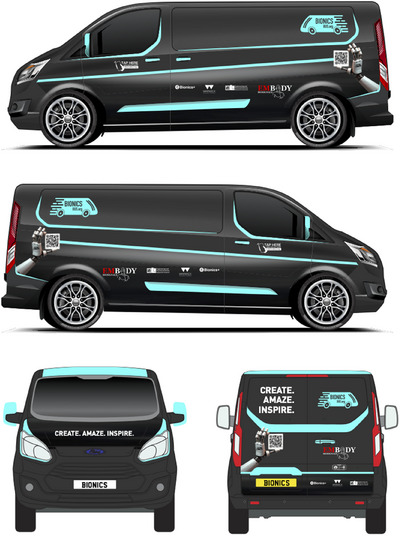
The Bionics Bus.

The Bionics Bus is a converted van that contains a number of experiments and devices based on the biomedical engineering field (Figure 2).

### 1.3 Clinical Service Delivery Using the Bionics Bus

The Bus can be used as a clinical service delivery tool for people with neurological, neuropsychiatric and psychiatric concerns [17]. This population is at high risk of digital exclusion given challenges of complexity in communication and cognitive deficits, multimorbidity and suitable digital templates focused on improving quality of life. Thus, innovative, cost‐effective approaches, such as the use of technology, are needed. The bus concept is to trial/deliver biotech solutions for treating neurological and neuro‐psychiatric conditions to populations that are at risk of disenfranchisement from clinical care due to digital exclusion. The Bus ambition is to see if it can deliver predictive (continuous monitoring of disease anticipates clinical events), personalised (responsive to individual needs rather than those of the population), preventative (Anticipates and reacts to the onset of adverse clinical events), participatory (ensuring they are able to take a proactive role in their own care) care knowledge.

We aimed to demonstrate the bus to the public to explore how it could be used to improve the accessibility, experience, and impact of neurology and neuropsychiatry care delivery and collect their views on it.

## 2 Methods

Our hypothesis is to assess the interest of the general public new emergent health technologies provided to their local communities in a mobile fashion i.e., through the Bionics Bus.

### 2.1 Study Design and Participants

The study followed Strengthening the Reporting of Observational Studies in Epidemiology (STROBE) checklist for reporting the cross‐sectional study [18]. It was conducted at the Annual Classic Motor Show at the Birmingham UK, National Exhibition Centre held from 8–10 November 2024. We defined the elements of our study design into a quantitative approach of identifying elements of *technology and healthcare*, *accessibility of healthcare systems* and *usefulness of Bionics Bus*. For the qualitative part we looked into categories of *relevance of technology and healthcare*, *the usefulness of Bionics Bus* and *the potential of Bionics Bus*.

### 2.2 Survey Tool (supplementary information 1)

A brief online survey (supplementary information 1) was developed by the study team to obtain the views of the general public. The survey was designed so that specific respondents would not be providing personal information that would render them identifiable. Informed consent to pool data and to publish was explicitly collected. Attendees were provided with information and video demonstrations relating to the functionality of the Bionics Bus, with a prototype of the bus also on display. Question items related to the demographic characteristics of the respondents (e.g., age group, ethnicity, citizenship, region of residence, level of education, current health concerns, familiarity with technology; access to healthcare and healthcare technology, thoughts on the Bionics Bus)and their views on implementation to increase healthcare technology‐driven accessibility, particularly on neurological and neuropsychiatric conditions were captured using 17 Likert questions with five responses and three open questions. This included barriers that they perceived the bus would encounter.

### 2.3 Ethics

Completion of the survey was regarded as consent to participate in the study and was explicitly mentioned as such.

### 2.4 Statistical Analysis

The survey employed convenience sampling using face‐to‐face discussion and providing a link to an online form for people to complete in their own time on their mobile/computer. The survey link was open for the duration of the show. The survey data from all participants was summarised descriptively. Additional analyses split the respondents into groups based on education and compare the responses between them.

### 2.5 Qualitative Analysis

Qualitative data for the three open questions, i.e. usefulness, utility and purpose of the bus was uploaded in NVivo 14 and thematically analysed (PT) following Braun and Clarke's (2006) six phases of thematic analysis [19].

## 3 Results

The responses of all the participants are detailed in Table 2; the table also shows a comparison of the responses of participants based on educational attainment. In order to facilitate the analysis, we will call the graduates group A and the A‐levels or less group, Group B.

**TABLE 2:** Responses of all participants and comparison of responses of participants based on educational attainment.

Question	Response (*n* = 54)	Group A: Graduates (*n* = 23)	Group B: A levels or lower (*n* = 28)
**Are you excited with what we are trying to do with the Bionics Bus? (Smiley Q)**	53 (98%)	22 (96%)	28 (100%)
**Participation consent**	52 (96%)	23 (100%)	28 (100%)
**Sex (M/F)**	37/15 (71%/29%)	14/9 (61%/39%)	22/6 (79%/21%)
**Age (years)**			
<18	1 (2%)	0	0
18–29	9 (17%)	7 (30%)	2 (7%)
30–50	22 (42%)	10 (44%)	12 (43%)
50+	20 (39%)	6 (26%)	14 (50%)
**Disability**			
None	33 (64%)	14 (61%)	20 (72%)
Neurodivergent or mental health	7 (14%)	3 (13%)	4 (14%)
Chronic physical health	10 (19%)	6 (26%)	4 (14%)
**Ethnicity**			
White	29 (56%)	14 (61%)	15 (54%)
Other	8 (15%)	4 (17%)	4 (14%)
Did not state	15 (29%)	5 (22%)	9 (32%)
**Nationality**			
British	48 (92%)	20 (87%)	27 (96%)
Non‐British	3 (6%)	2 (9%)	1 (4%)
Did not state	1 (2%)	1 (4%)	0
**How do you rate yourself and your capability with day‐to‐day technology?**			
Positive	33 (64%)	15 (66%)	16 (57%)
Neutral	16 (31%)	7 (30%)	11 (39%)
Negative	2 (4%)	1 (4%)	1 (4%)
**How enthusiastic are you to know more about new technologies, such as Artificial Intelligence (AI)?**			
Positive	40 (77%)	18 (78%)	20 (72%)
Neutral	9 (17%)	4 (17%)	6 (21%)
Negative	3 (6%)	1 (4%)	2 (7%)
**Do you struggle to access new information on technologies?**			
Yes Sometimes	11 (21%) 5 (10%)	5 (22%) 2 (9%)	6 (21%) 2 (7%)
No	36 (68%)	16 (69%)	20 (72%)
**How relevant do you feel technology should be in healthcare?**			
Positive	49 (94%)	22 (96%)	26 (92%)
Neutral	2 (4%)	1 (4%)	1 (4%)
Negative	1 (2%)	0	1 (4%)
**How accessible at present is Healthcare to you? (i.e., are clinics and hospitals easy to get to?)**			
Positive	21 (40%)	9 (39%)	11 (39%)
Neutral	18 (35%)	8 (35%)	10 (36%)
Negative	13 (25%)	6 (26%)	7 (25%)
**Do you get stressed attending routine health appointments? (please only answer this question if you don't have regular health check ups)**			
Yes	14 (28%)	6 (26%)	8 (29%)
Sometimes	19 (37%)	6 (26%)	12 (42%)
No	18 (35%)	10 (44%)	8 (29%)
**How useful do you think the Bionics Bus would be to deliver healthcare to you?**			
Positive	39 (75%)	17 (74%)	22 (79%)
Neutral	9 (17%)	5 (22%)	5 (17%)
Negative	4 (8%)	1 (4%)	1 (4%)
**Do you think the Bionics Bus will inspire young people to be interested in Science technology Engineering and Maths?**			
Positive	47 (90%)	21 (91%)	26 (93%)
Neutral	5 (10%)	2 (9%)	2 (7%)
Negative	0	0	0

Over the course of the event 54 people responded to the questionnaire overall, including 23 (43%) with a graduate background (Group A), 28 (52%) who had A‐level qualifications or lower (Group B), and 3 (6%) who did not report their non‐graduate/graduate status. The distribution of the identification by sex was a 71% (*n* = 37)/29% (*n* = 15) split between males and females overall, with a greater proportion of females represented in Group A (*n* = 9; 39%) compared to Group B (*n* = 6; 21%). Almost half the respondents (*n* = 22; 42%) were in the 30–50 years range, with 39% (*n* = 20) in the 50+ years range and 17% (*n* = 9) in the 18–29 years. range. Just 1 respondent (2%) was less than 18 years of age. Group A had a greater proportion of respondents in the 18–29 years age range (*n* = 7; 30%), whereas Group B had more in the 50+ years range (*n* = 14; 50%). The majority of respondents did not report any disabilities (*n* = 33; 64%), with 14% (*n* = 7) reporting neurodivergence and/or mental health, and 19% (*n* = 10) reporting having a chronic physical health disability. This ratio is approximately repeated for Group A (61%/13%/26%), whilst Group B featured a greater preponderance of respondents reporting no disability (*n* = 20; 72%), with less representation of those reporting chronic physical health issues (*n* = 4; 14%). Over half reported being of White ethnicity (*n* = 29; 56%) with 29% (*n* = 15) not stating their ethnicity—this is approximately mirrored across Groups A (*n* = 14; 61%) and B (*n* = 15; 54%). 92% (*n* = 48) of the respondents were of British nationality, again, this was approximately mirrored across Groups A (*n* = 20; 87%) and B (*n* = 27; 96%).

An overwhelming majority of respondents (*n* = 53; 96%) were excited with the proposal of the Bionics Bus and its intentions. Approximately two‐thirds of the respondents (*n* = 33; 64%) overall declared themselves as capable with day‐to‐day technology and only a minority (*n* = 2; 4%) as not capable—this was approximately mirrored across both Groups A (66%/4%) and B (57%/4%). There was a strong enthusiasm towards knowing more about new technologies (such as artificial intelligence), with 77% (*n* = 40) responding positively to this question item and only 6% (*n* = 3) showing a negative enthusiasm towards new technologies—this was approximately mirrored across both Groups A (78%/4%) and B (72%/7%). Over two‐thirds of respondents said that they do NOT struggle to access new information on technology (*n* = 36; 68%), whereas 10% (*n* = 5) reported that they sometimes struggle. This was approximately the same for both Group A (69%/22%) and Group B (72%/21%).

When asked about the relevance of technology in healthcare, the overwhelming majority of respondents were positive about the relevance (*n* = 49; 94%), with only around 2% (*n* = 1) reporting a negative view. This was approximately repeated in both Groups A (96%/0%) and B (92%/4%). When asked about the current accessibility of healthcare to them, only 40% (*n* = 20) reported that healthcare was currently accessible to them, with 35% (*n* = 18) showing a neutral view and a quarter of respondents (*n* = 13; 25%) reporting that healthcare was not accessible to them at present; this was similarly reflected in the responses of both Groups A (39%/35%/26%) and B (39%/36%/25%). When asked if respondents get stressed attending routine health appointments, almost two‐thirds reported either ‘yes’ or ‘sometimes’ (*n* = 33; 61%); with respondents from Group B more frequently reporting such stress (*n* = 20; 71%) compared to Group A (*n* = 12;55%).When linking the Bionics Bus to the delivery of healthcare, three quarters of respondents were positive about this (*n* = 39), with only 8% (*n* = 4) endorsing a negative response; these proportions were approximately reflected in both Groups A (74%/4%) and B (79%/4%).

Finally, when asked about the potential of the Bionics Bus with respect to inspiring young people to be interested in Science, Technology, Engineering, and Maths (STEM), the overwhelming majority (*n* = 47; 90%) were positive, with no negative responses (*n* = 0; 0%).

### 3.1 Qualitative Data Analysis

All participants expressed a positive perspective on the integration of technology into healthcare systems. Themes for all the qualitative analysis and direct quote examples are presented in Tables 3, 4 and 5 below. The full list of all comments is provided in SupplementaryMaterial 2. Participants highlighted that technology enhances accessibility, enabling patients to receive care more conveniently and efficiently, as reflected in the comment, “Better care, quicker care.” Participants also identified technology as synonymous with progress, emphasising its potential to drive innovation and advance healthcare delivery overall. The comments received underscored the important role of technology in shaping the future of healthcare, with participants stressing the importance of embracing new developments to remain competitive and responsive to emerging needs, as one noted, “We cannot be late to the game.” Overall, technology was perceived as a critical tool for the enhancement of healthcare systems, particularly in improving operational efficiency. It should also be noted that one participant commented on the ethical concerns around the use of technology in healthcare, commenting that “it should still be carried out by a qualified person.”

**TABLE 3:** Thematic analysis data with example of comments regarding relevance of technology in healthcare.

	Themes	Sample comments
1	Positive aspects of technology	
1.3	Enhancing Accessibility	Everyone should have available access Tech makes thinks easier and more effective
1.4	Technology is progress	Improve efficiency and reduce waiting times The world is moving to digitalised Technology advancement can help identify, prevent, and cure illness, and the better it gets, the better we can be

**TABLE 4:** Thematic analysis data with example of comments regarding what participants found useful (or not useful) with the Bionics bus.

	Themes	Sample comments
1	Useful aspects	Hope that technology can speed up diagnosis and treatments All the information provided by the team
1.3	Innovative and forward thinking	It looks cool and is very interesting Learning about the future opportunities
1.4	Accessible and inspiring	If it could be accessible to patients that struggle The accessibility of technology to help teach. I like that it is there to inspire young people in STEM
2	Room for improvement	Needs more explanation of what it can do Should be larger?

**TABLE 5:** Thematic analysis data with example of comments regarding the potential of the Bionics bus.

	Themes	Sample comments
1	Making a real difference	Amazing potential to make a real difference! This is just the beginning. The possibilities are endless
2	Accessibility is key	I see that it will support people and bridge the gap for people who need support and cannot get community support.
3	Education	Kids tend to love tech so hopefully it will attract them. Developing new ways to support local healthcare and inspire young people to persue STEM careers.
4	Improved healthcare	The future in diagnosis and treatment improvements. It can reduce waiting lists. Can keep people healthy.

*Note*: Please note that all quoted text is copied verbatim from the survey, with no grammatical/spelling errors corrected.

When asked about the perceived usefulness of the Bus, participants provided overwhelmingly positive feedback. The majority highlighted the innovative nature of the bus and the conceptual vision underpinning it, describing it as “inspired by future possibilities.” Participants also emphasised the Bus's role in improving healthcare accessibility, noting its capacity to “deliver healthcare to people who might not have access to it.” The prospect of quicker access to care was a source of excitement, and participants discussed its potential to inspire younger generations, suggesting it “will inspire future children to be scientific and to look above and beyond.”

Participants also acknowledged the importance of those managing and promoting the bus, recognising that “the staff were very enthusiastic,” which positively influenced public perception. While the general sentiment was favourable, some participants raised considerations for improvement. For example, one suggested that the bus should be larger, while another expressed uncertainty regarding “what it can do.”

When asked about the desired features and functions of the Bus, participants suggested that it should facilitate basic medical observations typically conducted by general practitioners. They also emphasized the importance of equipping the Bus with fundamental diagnostic tools, such as X‐ray portable machines, to expand its capabilities. Additionally, some participants expressed interest in integrating advanced technologies, particularly those related to health and AI, as they perceived these innovations to be both relevant and forward‐thinking. The provision of information services was also highlighted as a critical component, with some participants underscoring the value of therapeutic care options being made available on the Bus.

When discussing the potential of the Bus, participants responded with enthusiasm. They frequently emphasised the accessibility it could provide, particularly for patients in rural areas and those with limited mobility. Many participants reiterated its capacity to make a tangible difference and described it as offering “endless possibilities.” Additionally, the bus was recognised as a valuable educational tool, with participants noting its potential to serve as a “brilliant teaching resource for kids,” thereby fostering interest in healthcare and scientific innovation among younger generations.

Beyond educational benefits, participants highlighted the positive impact the bus could have on the healthcare system, including its ability to reduce waiting lists and contribute to “developing new ways to support local healthcare.” One participant also emphasised the potential for the bus to facilitate research, further underscoring its multifaceted value.

## 4 Discussion

There is a clear public health need for solutions for neurology and neuropsychiatry, and establishing public support for a proposed intervention is an important step towards this goal. Engaging communities is an important step to not only allow the public to acclimatise to potential significant changes but also to get them to express their confidence and concerns. It also provides an early opportunity for them to define the design of the proposed delivery of intervention.

We designed and delivered an online survey to gather the perspectives of the general public, and results obtained from the research are largely consistent with expectations. Looking ahead, there is an increasing reliance on technology, particularly with the integration of artificial intelligence, which is anticipated to play a pivotal role in this field. The findings of the research confirm that technological solutions are increasingly viewed as an indispensable aid across various sectors, including healthcare.

We used a mixed‐method approach, combining (i) quantitative as well as a (ii) qualitative approach to the study design, the outcomes can be summarised as:

- Quantitative (*technology and healthcare*, *accessibility of healthcare systems*, and *usefulness of Bionics Bus*): An overwhelming majority of respondents were completely supportive of the Bionics Bus idea, with a large majority supporting the view that evolving technology in healthcare is an important and inevitable step forward for their well‐being. A majority also reflected that they had problems accessing healthcare, and thus the Bionics Bus provides a solution to accessibility as well.
- Qualitative (*relevance of technology and healthcare*, *usefulness of Bionics Bus*, and *potential of Bionics Bus)*: There was an overall sense of positivity and indeed urgency in the introduction of healthcare technology through the Bionics Bus. In terms of its usefulness and potential, there was an overwhelming response indicating the timeliness of a system that could address the accessibility problem of healthcare and which showed such potential in its flexibility of approach.

Overall, people were positive about receiving care in their own communities using technology. The respondents, across age range and educational status, were open to new technology being a vehicle of care delivery. There was a recognition that there are currently challenges in the current care delivery mechanisms, with a significant number of respondents finding it difficult and/or challenging to access clinical services.

A limitation of this study is that the respondent population may not be entirely representative of the United Kingdom general population, with a high proportion of male respondents (71%). Additionally, they may conceivably be more likely to endorse a transport‐based solution to healthcare problems relative to the general population, considering their presumed baseline interest in motor vehicles by virtue of their attendance at a Motor Show. A strength of this study is that data has been obtained regarding the public perception of a novel outreach intervention for hard‐to‐reach patients with neuropsychiatric problems.

## 5 Conclusion

This project was a template/feasibility on how to link with people to understand the delivery of new frameworks of care, such as taking technologies to local communities. Further research needs to explore further the component parts of a Bionics Bus that would yield the greatest level of healthcare benefit to otherwise hard‐to‐reach patients with neuropsychiatric problems, as well as a corresponding economic analysis to estimate the financial viability of such an intervention.

## Author Contributions

**Christopher James**: conceptualisation, formal analysis, funding acquisition, investigation, project administration, supervision, validation, visualisationand writing – original draft. **Sarang Shankar**: conceptualisation, formal analysis, investigation, validation, visualisationand writing – original draft. **Samuel J Tromans**: formal analysis, investigation, validation, visualisation, writing – review and editing. **Richard Laugharne**: Conceptualisation, formal analysis, investigation, project administration, supervision, validation, visualisation, writing – review and editing. **Paraskevi Triantafyllopoulou**: formal analysis, investigation, project administration, validation, visualization, writing – review and editing. **Maria Richards**: conceptualisation, investigation, visualisation, writing – review and editing. **Rohit Shankar**: conceptualisation, data curation, formal analysis, funding acquisition, investigation, methodology, project administration, resources, supervision, validation, visualisation, writing – original draft, review and editing.

## Conflicts of Interest

Christopher James is the developer and inventor of the Bionics Bus, the product discussed significantly in the paper. RS has received institutional and research support from LivaNova, UCB, Eisai, Veriton Pharma, Neuraxpharm, Bial, Angelini, UnEEG and Jazz/GW pharma outside the submitted work. No other author has any declared conflict of interest related to this paper. SJT has received research support from the Baily Thomas Charitable Fund, the Wellcome Trust, NIHR, NHS Digital and Jazz Pharma.

## Supporting information



Supporting Information

Supporting Information

## Data Availability

All data used for the paper is within the manuscript.

## Appendix 1 Description of the Bionics Bus Concept

### 1.1

The Bionics Bus is a modern, ‘spaceship’‐styled vehicle—both inside and out (Figure 2). When the bus arrives on the scene, the bus crew will set up, the back door will open revealing a big screen, and the side door will slide open showing a ‘cockpit‐like' interior. Depending on the reason behind the visit, a volunteer is seated, and one of the Bus crew goes through a script showing the volunteer how their health and well‐being is being monitored rightnow—in fact, they will see a whole raft of data: they will be able to communicate with devices using eye‐tracking and brain‐computer interfacing technologies, as well as having vital signs monitored and displayed. Whilst the volunteer goes through the script, the rest of the bus crew are describing what is going on inside to the crowd outside through the big screen display. They can also demonstrate smaller handheld tech and experiments and hand out information fliers, as well as answer questions put to them.

## References

[1] J. D. Steinmetz , K. M. Seeher , N. Schiess , et al., “Global, Regional and National Burden of Disorders Affecting the Nervous System, 1990–2021: A Systematic Analysis for the Global Burden of Disease Study 2021,” The Lancet Neurology 23, no. 4 (2024): 344–381. 38493795 10.1016/S1474-4422(24)00038-3 PMC10949203

[2] NHS England , “ Five Year Forward View ,” (2014), https://www.england.nhs.uk/wp‐content/uploads/2014/10/5yfv‐web.pdf.

[3] A. Nitkunan , J. Lawrence , and M. Reilly , “Neurology Workforce Survey Association of British Neurologists 2018–2019,” (2020), https://cdn.ymaws.com/www.theabn.org/resource/collection/219B4A48‐4D25‐4726‐97AA‐0EB6090769BE/2020_ABN_Neurology_Workforce_Survey_2018‐19_28_Jan_2020.pdf.

[4] “ Number of Psychiatrists: How do Countries Compare? Eurostat ,” (May, 2020), https://ec.europa.eu/eurostat/web/products-eurostat-news/-/ddn-20200506-1#:~:text=The%20EU%20countries%20with%20the,inhabitants%3B%20Sweden%202016%20data).

[5] The Neurological Alliance , “ The Invisible Patients: Revealing the State of Neurology Services .” (January 2015), https://www.neural.org.uk/assets/pdfs/2015-01-invisible-patients.pdf.

[6] Chief Medical Officer , “ Chief Medical Officer's Annual Report 2021 Health in Coastal Communities ,” (July 2021), https://www.gov.uk/government/publications/chief-medical-officers-annual-report-2021-health-in-coastal-communities.

[7] Public Health England , “ Health and Wellbeing in Rural Areas ,” (March 2017), https://www.local.gov.uk/publications/health-and-wellbeing-rural-areas.

[8] A. Nitkunan , J. Lawrence , and M. M. Reilly , “Association of British Neurologists: UK Neurology Workforce Survey′, ACNR. (2020)23. 26.The Neurological Alliance. The Invisible Patients: Revealing the State of Neurology Services,” Advances in Clinical Neuroscience and Rehabilitation 20, no. 1 (2020): 28–32, https://www.neural.org.uk/assets/pdfs/2015-01-invisible-patients.pdf.

[9] Public Health England , “ Deaths Associated with Neurological Conditions in England 2001 to 2014 ,” (2018), https://assets.publishing.service.gov.uk/government/uploads/system/uploads/attachment_data/file/683860/Deaths_associated_with_neurological_conditions_data_analysis_report.pdf.

[10] NIHR , “ *Rehabilitation: How can Services Meet Demand*?,” (September 2021), https://evidence.nihr.ac.uk/collection/rehabilitation-how-can-services-meet-demand/.

[11] A. Cieza , K. Causey , K. Kamenov , S. W. Hanson , S. Chatterji , and T. Vos , “Global Estimates of the Need for Rehabilitation Based on the Global Burden of Disease Study 2019: A Systematic Analysis for the Global Burden of Disease Study 2019,” Lancet (London, England) 396, no. 10267 (2021): 2006–2017, 10.1016/S0140-6736(20)32340-0. 33275908 PMC7811204

[12] D. T. Wade , “The Future of Rehabilitation in the United Kingdom National Health Service: Using the COVID‐19 Crisis to Promote Change, Increasing Efficiency and Effectiveness,” Clinical Rehabilitation 35, no. 4 (2021): 471–480, 10.1177/0269215520971145. 33167682

[13] A. Kennedy , D. Reeves , P. Bower , et al., “The Effectiveness and Cost Effectiveness of a National Lay‐Led Self Care Support Programme for Patients With Long‐Term Conditions: A Pragmatic Randomised Controlled Trial,” Journal of Epidemiology and Community Health 61, no. 3 (2007): 254–261, 10.1136/jech.2006.053538. 17325405 PMC2652924

[14] K. Lorig , P. L. Ritter , F. J. Villa , and J. Armas , “Community‐based Peer‐Led Diabetes Self‐Management: A Randomized Trial,” The Diabetes Educator 35, no. 4 (2009): 641–651, 10.1177/0145721709335006. 19407333

[15] Darzi, A. “*Investigation of the National Health Service in England*,” (September, 2024), https://assets.publishing.service.gov.uk/media/66e1b49e3b0c9e88544a0049/Lord-Darzi-Independent-Investigation-of-the-National-Health-Service-in-England.pdf.

[16] C James , “ Bionics Bus ,” (November, 2024), https://www.BionicsBus.org.

[17] WHO , “ ICD‐11 Classification ,” (2021), https://icd.who.int/en.

[18] E. von Elm , D. G. Altman , M. Egger , et al., “The Strengthening the Reporting of Observational Studies in Epidemiology (STROBE) Statement: Guidelines for Reporting Observational Studies,” Journal of Clinical Epidemiology 61, no. 4 (2008): 344–349, 10.1016/j.jclinepi.2007.11.008. 18313558

[19] V. Braun and V. Clarke , “Using Thematic Analysis in Psychology,” Qualitative Research in Psychology 3, no. 2 (2006): 77–101, 10.1191/1478088706qp063oa.

