# Supplementary material for: The Bionics Bus for Neurology and Neuropsychiatry: Concept Development and Validation
Source: Healthc Technol Lett. 2025 Mar 20;12(1):e70008. doi: 10.1049/htl2.70008 (PMC11926247; doi:10.1049/htl2.70008)
Supplement: Supplementary file 1 — Supporting Information [file HTL2-12-e70008-s002.pdf]

The Bionics Bus

Our mission is to excite! We want to reach people of all ages and spread the word about Science, Technology, Engineering, Maths as well as Digital and research Healthcare. The Bionics Bus is jam packed with biomedical engineering technology. The Bus can come to you and you can interact with the advanced technology and with the investigators in the Bus. We need to understand from you

A. How useful do you think the idea is?

B. What do you think should be in the Bionics Bus?

This survey will take 5 minutes of your time. All your answers will be anonymous. We would look to publish the summary findings of this survey in a scientific journal. Answering our questions will be considered informed consent. Thank you for your help and contribution in making a difference to society!

* Indicates required question

1. **Are you excited with what we are trying to do with the Bionics Bus?** *

Mark only one oval.

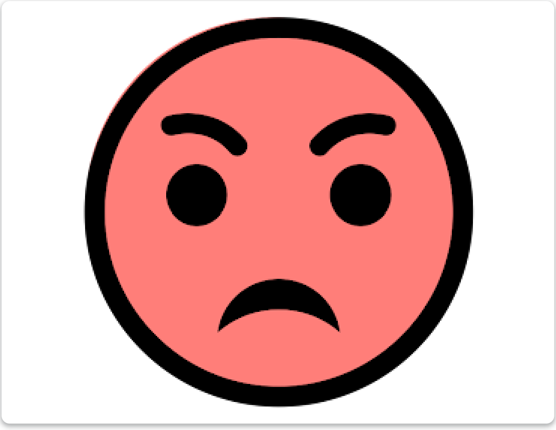

- Really not!

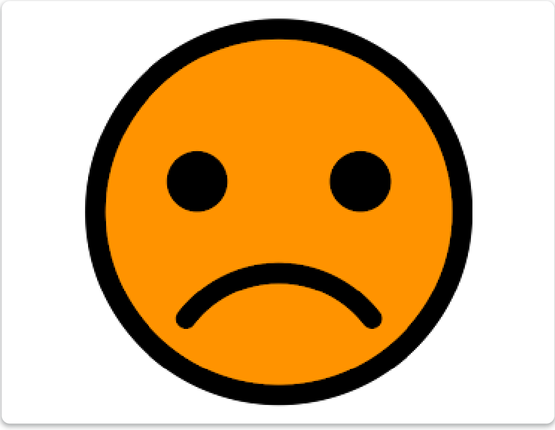

- Not!

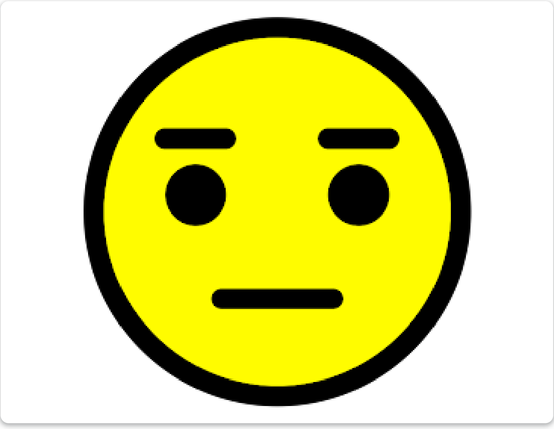

- Indifferent

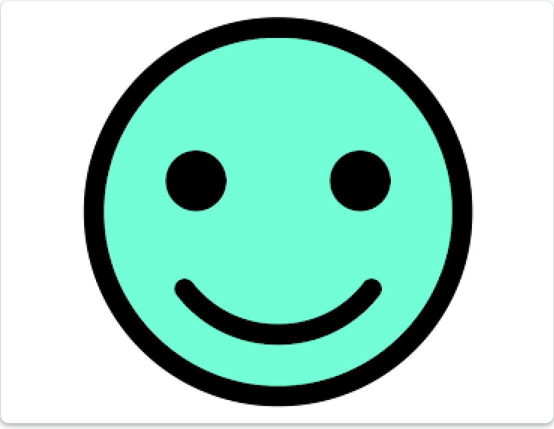

- Excited

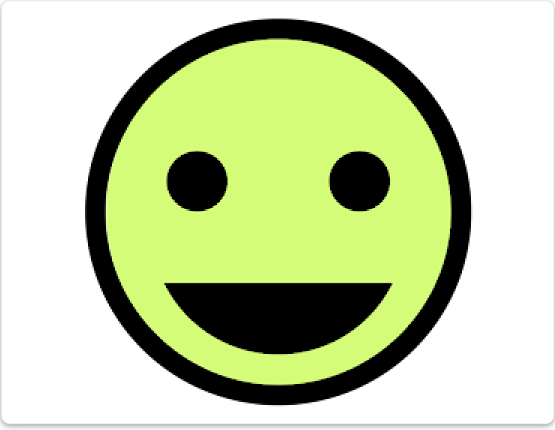

- Really excited

Happy to help?

2. Are you happy for your information to be pooled anonymously and scientifically published to help gain funding and further develop the bus? *

Mark only one oval.

- YES
- NO

About You

3. How do you identify yourself as?

Mark only one oval.

- Male
- Female
- Other
- Prefer not to say

4. Age

Mark only one oval.

- Under 18
- 18 to 29
- 30 to 50
- Over 50
- Prefer not to say

5. Do you have any special needs? *(tick more than one if needed)*

Check all that apply.

- [ ] Chronic physical health problems (including mobility)
- [ ] Mental health problems
- [ ] Neurodiversity including Autism/ADHD etc.
- [ ] Other
- [ ] None
- [ ] Prefer not to say

6. Ethnicity - please add your ethnicity

____

7. Nationality

Mark only one oval.

- British
- Non-British
- Prefer not to say

8. If resident in UK please could you share your first part of the postcode for us to understand the urban: rural divide

____

9. Your highest level of education is:

Mark only one oval.

- Non- GCSE
- GCSE level
- A levels/equivalent
- Bachelor Degree
- Post Graduate qualification

About You and Technology

10. How do you rate yourself and your capability with day to day technology?

Mark only one oval.

	1	2	3	4	5	
Poo	◯	◯	◯	◯	◯	High

11. How enthusiastic are you to know more about new technologies, such as Artificial Intelligence (AI)?

Mark only one oval.

	1	2	3	4	5	
Not	◯	◯	◯	◯	◯	Very interested

12. Do you struggle to access new information on technologies?

Mark only one oval.

- ◯ YES
- ◯ NO
- ◯ Other: ____________________

13. How relevant do you feel technology should be in Healthcare?

Mark only one oval.

	1	2	3	4	5	
Mini	◯	◯	◯	◯	◯	Highly relevant

14. What was the reason for your choice above?

____

15. How accessible at present is Healthcare to you? (i.e. are clinics and hospitals easy to get to?)

Mark only one oval.

	1	2	3	4	5	
Very	◯	◯	◯	◯	◯	Not at all accessible

16. Do you get stressed attending routine health appointments? (please only answer this question if you don't have regular health check ups)

Mark only one oval.

	1	2	3	4	5	
Not	◯	◯	◯	◯	◯	Very stressed

About You and the Bionics Bus

17. How useful do you think the Bionics Bus would be to deliver Healthcare to you?

Mark only one oval.

	1	2	3	4	5	
Not	◯	◯	◯	◯	◯	Very useful

18. Do you think the Bionics Bus will inspire young people to be interested in Science technology Engineering and Maths?

Mark only one oval.

	1	2	3	4	5	
Not	◯	◯	◯	◯	◯	Absolutely!

19. What did you find useful (or not useful) with the Bionics Bus?

____

20. What do you think should be on the Bionics Bus, and why?

____
____
____
____
____

21. What do you see the potential for the Bionics Bus?

____

This content is neither created nor endorsed by Google.

Google Forms
