# Supplementary material for: The Bionics Bus for Neurology and Neuropsychiatry: Concept Development and Validation
Source: Healthc Technol Lett. 2025 Mar 20;12(1):e70008. doi: 10.1049/htl2.70008 (PMC11926247; doi:10.1049/htl2.70008)
Supplement: Supplementary file 2 — Supporting Information [file HTL2-12-e70008-s001.docx]

| **Reason for opinion on technology in healthcare** |
| --- |
| Accessibility |
| Enhanced monitoring |
| Important |
| The future |
| As it can tell us what’s wrong and what to do |
| None-invasive procedure |
| Technology is very important for progress. |
| Technology can impart high levels of knowledge, AI can diagnose much quicker than a single individual. |
| Work in healthcare |
| Tech makes thinks easier and more effective |
| Improve efficiency and reduce waiting times |
| Doctors should read what is not being said. And ask the patients "how is everything else in your life " read the face, read the mobility etc. |
| Technology will only make our health and healthcare services better |
| I currently work within IT at a big tech firm |
| Too long waiting lists |
| Difficult to see a doctor |
| iMPORTAT as signce is needed |
| The more help technology can give a patient the better. |
| Technically is everywhere in todays like I struggle with it as I’m the old school who worked with my hands |
| The world is moving to digitalised |
| It helps |
| Healthcare needs to innovate |
| Makes diagnosis easier |
| It will improve efficiency |
| Important isn’t it |
| Better care quicker care |
| Labour saving and diagnosis |
| We cannot be late to the game |
| It can aid humans greatly and can support improvements. |
| It should still be carried out by a qualified person due to mutations in virus and Illness |
| It's useful |
| Demographics |
| times need to change and move forward. technology is extremely important in that |
| Technology brings innovation and improvement |
| Everyone should have available access |
| It’s the way of the future |
| Everything is moving online |
| Technology if used correctly could benefit many people |
| New tech is going a long way |
| Technology advancement can help identify, prevent, and cure illness, the better it gets, the better we can be. |
| I believe in some area's technology is important but so is face to face consultations |
| I need it |
| It’s the future and easier to diagnose and treat |
| **What did you find useful (or not useful) with the Bionics Bus?** |
| Forward thinking |
| Its ability to deliver healthcare to people who might not have access to it |
| Portable |
| It looks cool and is very interesting |
| Access any ware |
| Inspired by the future possibilities of such an inspiring. |
| Discussion with the team |
| Advertising technology |
| Classic car show |
| Nothing, |
| The concept is amazing and the younger generations are screaming out for it |
| needs more explanation of what it can do |
| It can help me keep calm |
| should be larger? |
| The accessibility of technology to help teach. |
| Training for young people is essential not everyone has the ability to attend university but still deserve a chance in life |
| Interesting concept |
| Learning about the future opportunities |
| My memory |
| Talking to experts |
| Quick appointments |
| Quicker care |
| Hope that technology can speed up diagnosis and treatments |
| Technology |
| Everything all in one place and will inspire future children to be scientific and to look in above and beyond. |
| The technical side |
| The staff were very enthusiastic |
| The usage of AI technology to provide diagnosis |
| Information |
| All the information provided by the tesm |
| Well presented and interactive |
| Very useful |
| I like that it is there to inspire young people in STEM |
| If it could be accessible to patients that struggle |
| I struggle with my mental health but my GP cannot help nor any of the mental health team |
| Na |
